# Comparison of two planning systems for HDR brachytherapy gynecological application

**DOI:** 10.1120/jacmp.v2i3.2604

**Published:** 2001-09-01

**Authors:** Osman A. Elhanafy, Mostafa D. Migahed, Hanim A. Sakr, Mostafa Ellithy, Rupak K. Das, Heath J. Odau, Bruce R. Thomadsen

**Affiliations:** ^1^ Department of Human Oncology University of Wisconsin Madison Wisconsin 53792; ^2^ Mansoura University Egypt; ^3^ Department of Human Oncology University of Wisconsin Madison Wisconsin 53792 Egypt; ^4^ Departments of Medical Physics and Human Oncology University of Wisconsin adison Wisconsin 53792

**Keywords:** HDR Brachytherapy, gynecological brachytherapy, radiotherapy treatment planning

## Abstract

**Purpose**: This report compares the Nucletron NPS and PLATO planning system for patients treated for cervix cancer.

**Materials and Methods**: This study compares calculations generated using the older NPS (version 11.43) planning system and the more recent PLATO (version 14.1) system for two cases: 1) a single dwell position and 2) an actual patient application using a tandem and ovoid.

**Results**: *For one dwell position:* for NPS planning the dose for points along the source axis forward of the cable was 9.85% more than for symmetrically placed points in the cable direction. For PLATO, the same test gave rise to a difference of 10.2%. Comparing the two systems, NPS calculated doses for points in the forward direction 14% greater than those calculated by PLATO. The entry of points using the digitizer accounted for less than 1% of any difference. *For the patient case:* the dose difference between NPS and PLATO planning for all patient reference points entered from films ranged from 1 to 4%. The difference in dose between optimized and nonoptimized planning was approximately 0.5% for prescription points (points *A*), while for the bladder and rectum the differences were 6% and 20%, respectively with NPS, and with PLATO, 8% and 22%, respectively.

**Conclusion**: This study highlighted the effects of the differences in the calculational algorithm between the older and newer planning systems from Nucletron. While the differences were minimal on the perpendicular bisector of the source, along the axis they become considerable. In a practical gynecological case, these differences mostly affect the dose to the rectum, since that organ receives the greatest proportion of its dose from rays near the same axis. Overall, the PLATO system plan required about 2.5% less integrated reference air kerma than the NPS plan for the same dose to point *A*. For either planning system, optimization is crucial in decreasing dose to bladder and rectal points. © *2001 American College of Medical Physics.* [DOI: 10.1120/1.1384528]

PACS number(s): 87.53.–j

## INTRODUCTION

The manufacturer of one common high dose rate (HDR) brachytherapy unit recently released a new treatment planning system. This report compares dose distribution calculated with the older and the newer treatment planning systems.

## MATERIALS AND METHODS

### I. Dose Calculation

High dose rate (HDR) brachytherapy with the Nucletron microSelectron (Nucletron Corp, Columbia, MD) uses a single ${}^{192}\text{Ir}$ source with an active dimension of 3.5 mm long by 0.5 mm in diameter, in a steel capsule 6 mm long by 1.1 mm diameter and has a half‐life of 74 days.^1^ The source is maintained with an activity between approximately 150 and 370 GBq (4 and 10 Ci), and is attached to a computerized drive mechanism used to move the source to the predetermined dwell position within the application catheter. The two treatment planning systems both run on the same Unix‐based Silicon Graphics (Silicon Graphics, Mountain View, CA) computer. The older system, NPS (version 11.43) is a menu‐driven program, while PLATO is window based. Both the input and display of planning use a single monitor. In NPS and PLATO planning, a digitizer is used to input the positions of sources and some of the dose calculation points.

**Figure 1:**
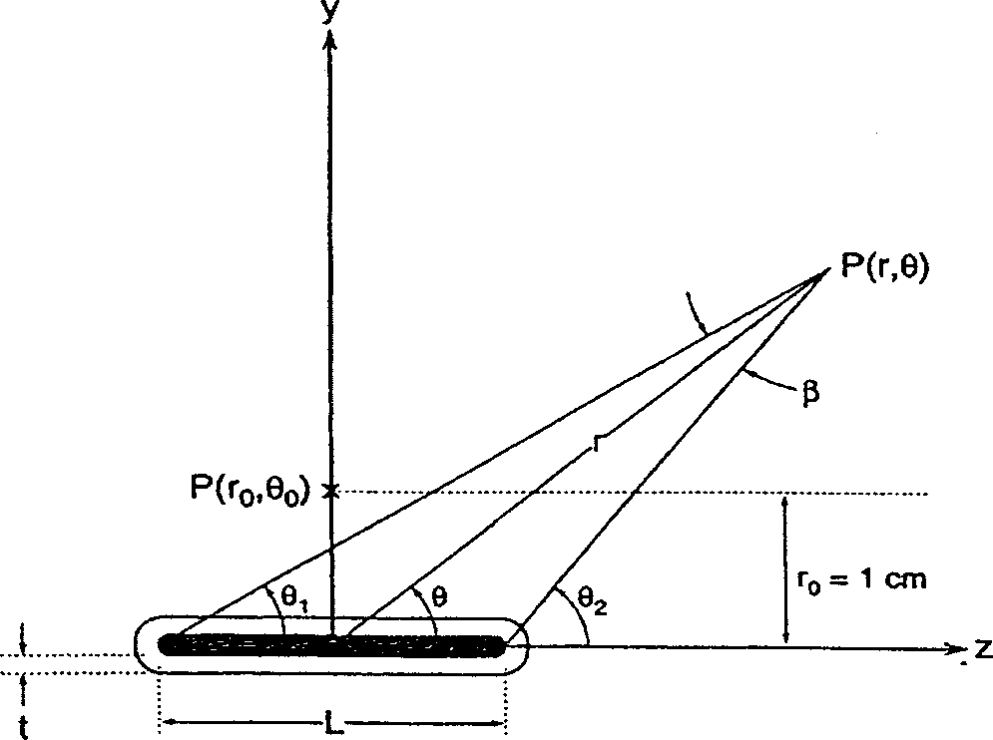
Geometry assumed in the dose calculation formalism. Angle *β* is that subtended by the active length at point *P*.

The dose‐calculation algorithm of the PLATO planning system is based on the recommendations of AAPM Task Group 43.^2^ The dose distribution can be described in terms of a polar coordinate system with origin at the source center. According to this formalism, the dose rate distribution around a brachytherapy source can be written as

(1) $$\dot{D}(r,\theta )=S_{K}\Lambda [G(r,\theta )/G(r_{0},\theta_{0})]g(r)F(r,\theta ),$$

where $S_{K}$ is air kerma strength of the source, Λ is the dose rate constant, *G*(*r*, *θ*) is the geometry factor, *g*(*r*) is the radial dose function, *F*(*r*, *θ*) is the anisotropy function, and *r* is the distance to the point of interest and *θ* is the angle with respect to the long axis of the source as illustrated in Fig. 1.

The dose calculation of NPS is based on the conventional approach. The dose rate *D*(*r*, *θ*) in tissue at point *P*(*r*, *θ*) is given by,

(2) $$\dot{D}(r,\theta )=A_{a}{\left(\Gamma_{\delta }\right)}_{x}f[\beta /(L\sin\theta )]T(r)F(\theta ),$$

where $A_{a}$ is the apparent activity, ${\left(\Gamma_{\delta }\right)}_{x}$ is the exposure rate constant, *f* is the exposure to dose conversion factor, *β* is the angle subtended by the active length at point *P*, *θ* is the angle between the ray to point *P* with the source axis, *L* is the active length of the source, *T*(*r*) is the tissue attenuation and scatter correction function, and *F*(*θ*) is an anisotropy correction that is only dependent on angle *θ*.

The NPS program used a calculation grid spacing of 1 mm. The grid spacing for PLATO is adjustable and depends on the magnification of the image on the screen. In this study, the default values for the calculation grid were used, which approximated a grid spacing of 0.5 mm.

### II. Geometrical Arrangement

To evaluate the calculational aspects of the Nucletron planning systems, two cases were investigated. The first was a single dwell position. The second case study used the geometry of an actual patient treated using a tandem and ovoids. This latter application used 22 dwell positions. The 6.5‐cm tandem contained 14 dwell positions and the ovoids each contained four. All dwell positions were separated by 5 mm. This case included dose calculations to prescription points (point *A*, right and left) and patient reference points (bladder, rectum, and points *E, B*, and *P*, right and left). Point *A* is 2 cm lateral perpendicular to the midline of the intrauterine canal and 2 cm cephalad along the tandem from the external cervical os.^3^ ^,^ ^4^ Point *B* and *P* lie 2 cm cephalad of the external os along the body axis 5 and 6 cm, respectively, to the right and left of the patient's midline in the transverse plane. The bladder and rectal points are in accordance to the definition of ICRU Report 38.^5^ Point *E* right lies at the intersection of a line tangent to the superior aspect of the acetabula and a line tangent to the medial aspect of the right acetabulum. Point *E* left is similarly defined.

## RESULTS AND DISCUSSIONS

### I. Single Dwell Position

**Figure 2:**
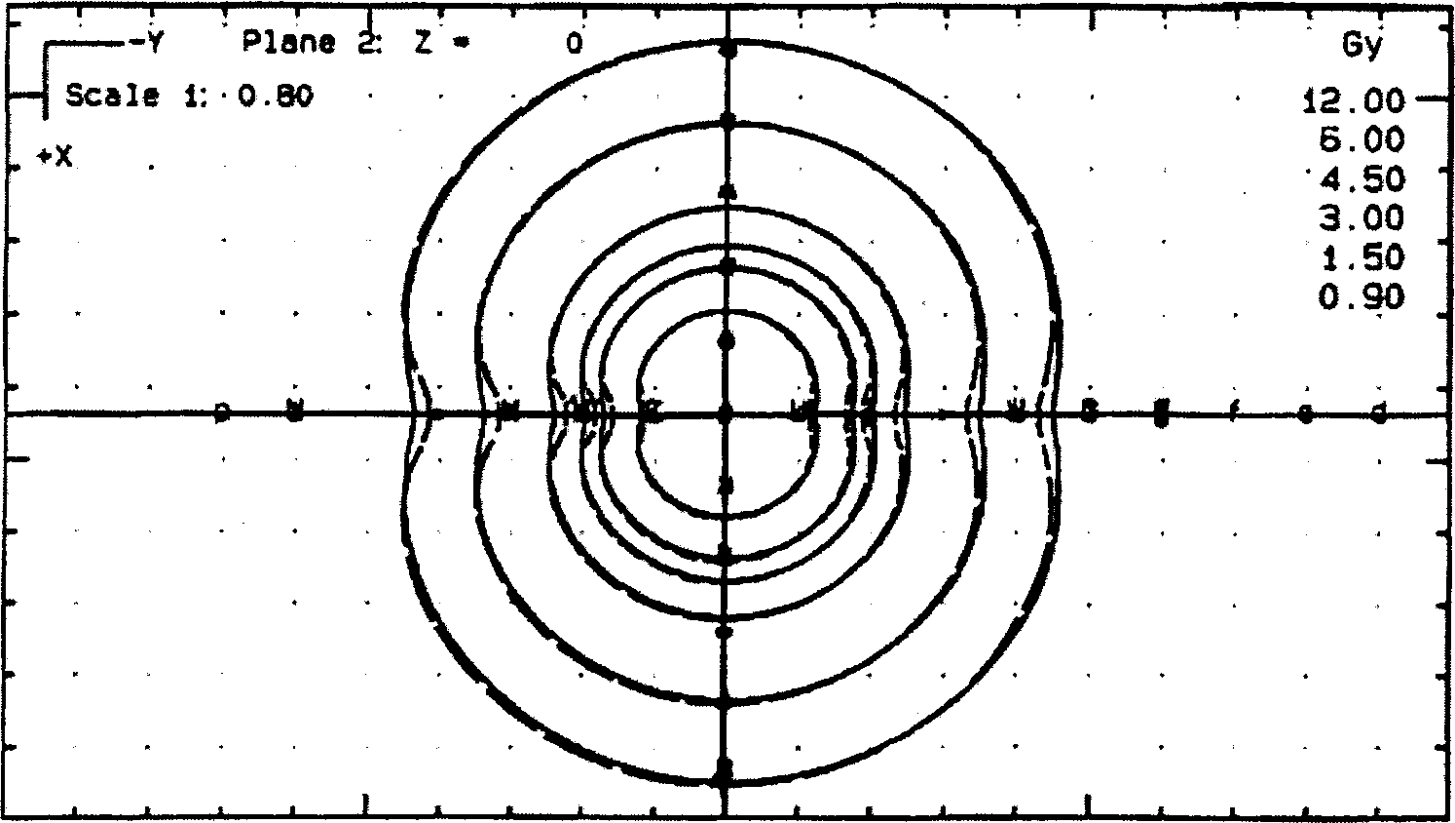
Comparison between PLATO (dash lines) and NPS (solid lines) isodose lines for 200, 100, 75, 50, 25, and 15% of the prescription point for single dwell position. The figure also shows the position of reference points used for the comparison illustrated in Fig. 3.

Figure 2 shows the differences in the isodose line distributions for 200, 100, 75, 50, 25, and 15% of the prescription point of 6 Gy, generated by PLATO and NPS. The greatest differences are along the source axis due to the different anisotropy functions used by the two programs. The dose was calculated at 40 reference points specified by coordinates with 10 points each on both sides of the perpendicular bisector and 10 each along both sides of the axis.

**Figure 3:**
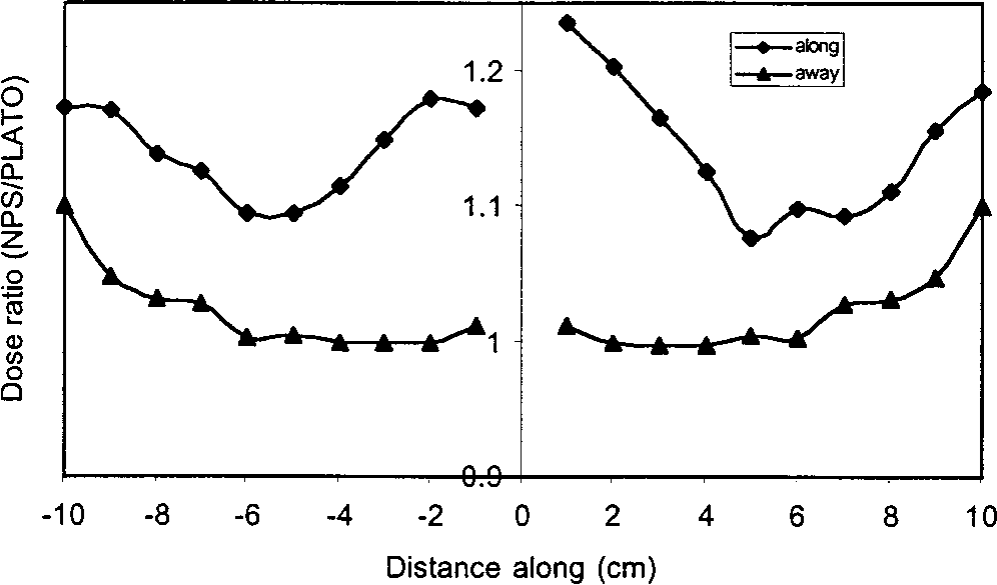
The ratio of the dose calculated for reference points along from and along the source axis for NPS and PLATO for a single dwell position.

Figure 3 illustrates the ratio of the dose (NPS/PLATO) calculated for points along the source axis for NPS and PLATO. For NPS planning, the dose for points forward of the cable along the same axis was 9.85% more than the calculated dose for symmetrically placed points in the cable direction. For PLATO, the same test gave rise to a difference of 10.2%. The mean difference in the dose calculated in the forward direction by NPS and Plato was 14% (NPS higher), while in the cable direction the mean difference was 14.5%.

Figure 3 also illustrates that along the perpendicular bisector there was little difference between the doses to points, out to 9 cm. The mean difference between NPS and PLATO in this direction was 2.3%. The differences represent the variation in factors for attenuation and scatter for the two models. To evaluate the significance of these differences compared with the input uncertainty, the doses for each system were compared using data entry by the digitizer and by manual coordinate entry. The difference between the dose to points, following digitizer and manual entry, was less than 1%.

### II. Patient Case

**Figure 4:**
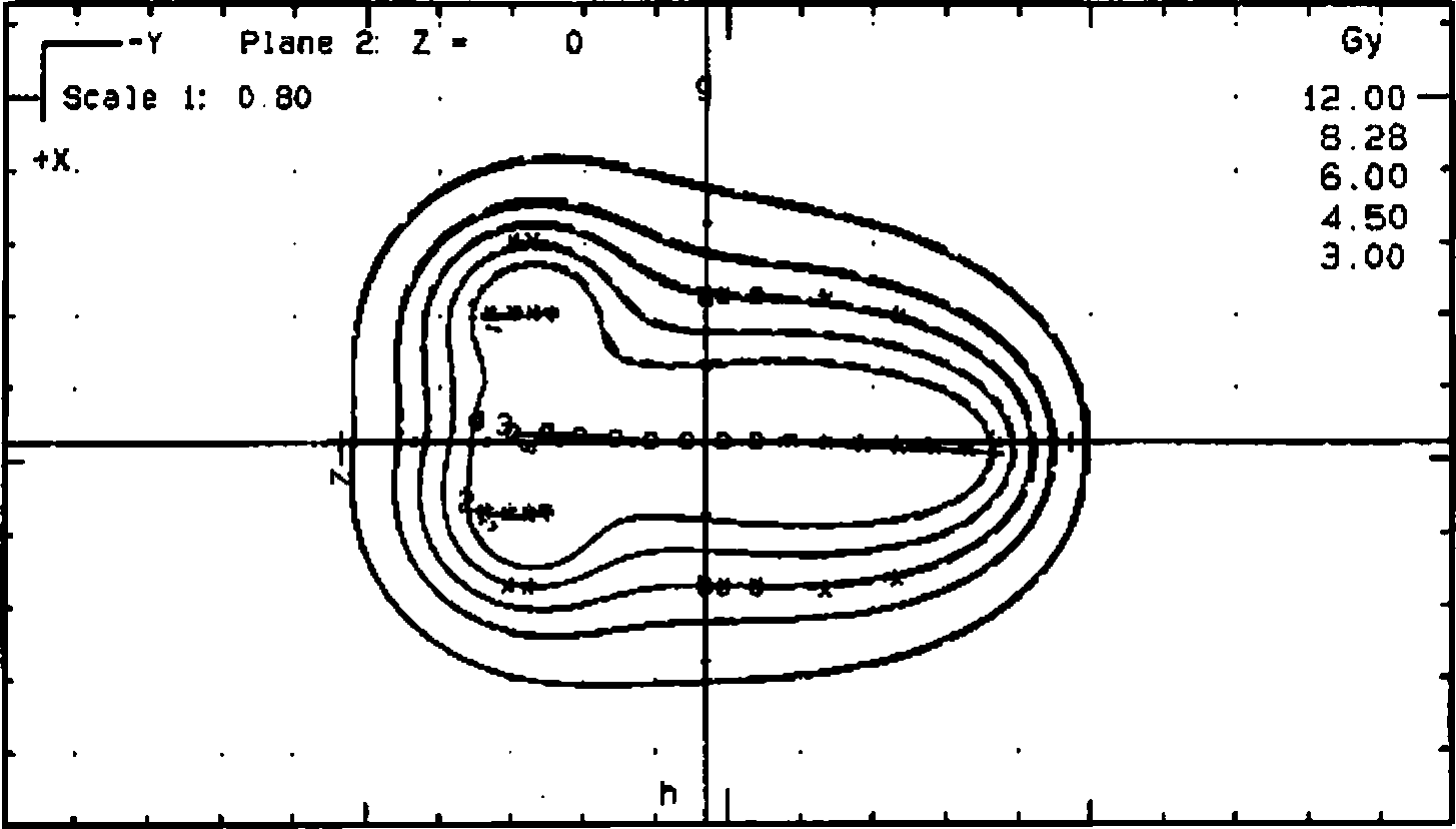
Comparison between PLATO isodose lines optimized (dash lines) and NPS (solid lines) isodose lines optimized, which were 200, 138, 100, 75, and 50% of the prescription point by using 22 dwell positions.

Table I illustrates calculated dose (Gy) using NPS and PLATO planning for prescription points (point *A*, right and left) and patient reference points (bladder, rectum, and points *E, B*, and *P*, right and left). These data correspond to dwell times optimized to deliver specified doses to the set of optimization points, as shown in Fig. 4, along the tandem and lateral to the ovoids. The data resulted from point optimization using a dwell time gradient factor equal to 0.5 and based on 100% of the mean dose to the applicator points (taken as points *A*). There is no difference between NPS and PLATO planning for the dose to the prescription points. The dose difference between NPS and PLATO planning for all reference points ranged from 1 to 4%.

**Table I:** Calculated dose (Gy) to reference and prescription points for the real clinical case using the NPS and PLATO planning optimized and nonoptimized.

	Dose (Gy)			Dose (Gy)		
Points	NPS optimized	NPS nonoptimized	Ratio	PLATO optimized	PLATO nonoptimized	Ratio
Right *A*	6.14	6.14	1.00	6.14	6.17	1.00
Left *A*	5.86	5.79	0.99	5.86	5.82	0.99
Bladder	2.36	2.51	1.06	2.38	2.56	1.08
Rectum	5.02	6.02	1.20	4.96	6.07	1.22
Right *E*	0.84	0.81	0.96	0.82	0.78	0.95
Left *E*	0.84	0.82	0.98	0.81	0.8	0.99
Right *B*	1.74	1.75	1.01	1.73	1.75	1.01
Left *B*	1.59	1.61	1.01	1.58	1.61	1.02
Right *P*	1.23	1.23	1.00	1.21	1.22	1.01
Left *P*	1.17	1.18	1.01	1.16	1.17	1.01

Table I also shows the doses to the same points using the same dwell position geometry, but assigning a constant dwell time to all positions (nonoptimized application). The difference in dose between NPS and PLATO planning for the prescription points (point *A*, right and left), is small, approximately 1%, and for all reference points, again from 1 to 4%.

Figure 4 illustrates a small difference between optimized isodose lines, which were 200, 138, 100, 75, and 50% of the prescription points (6 Gy), for NPS and PLATO programs.

The mean difference in dose between NPS optimized and NPS nonoptimized, which is the same dwell time for all dwell positions, for the prescription points was approximately 0.5%. However, the differences for bladder and rectum were 6% and 20%, respectively. The difference in dose to points *E, B*, and *P* remained less than 1%, since they fall relatively far from the applicator. For the PLATO planning system, the differences in dose between optimized and nonoptimized planning for the prescription points is 1%; bladder and rectum was 8% and 22%, respectively; and points *E, B*, and *P* were 3%, 1.5%, and 1%, respectively. These data show that the optimization is very important to minimizing the dose to the bladder and rectum with the tandem and ovoids applicator. Table II illustrates the dose (Gy) calculated in NPS, manually entering the results from optimization using the PLATO. The difference in dose between NPS optimized dwell times and those from PLATO was 2.45% for point *A*; 2.6% bladder; 3.3% rectum; and for points *E, B*, and *P* was 1.8%, 2.4%, and 2.1%.

**Table II:** Calculated dose (Gy) to reference and prescription points for the real clinical case HDR using the dwell time from PLATO in NPS.

	Dose (Gy)		
Points	NPS	PLATO in NPS	Ratio
Right *A*	6.22	6.07	1.025
Left *A*	5.93	5.79	1.024
Bladder	2.39	2.33	1.026
Rectum	5.08	4.92	1.033
Right *E*	0.85	0.84	1.012
Left *E*	0.85	0.83	1.024
Right *B*	1.76	1.72	1.023
Left *B*	1.61	1.57	1.025
Right *P*	1.24	1.22	1.016
Left *P*	1.19	1.16	1.026

**Figure 5:**
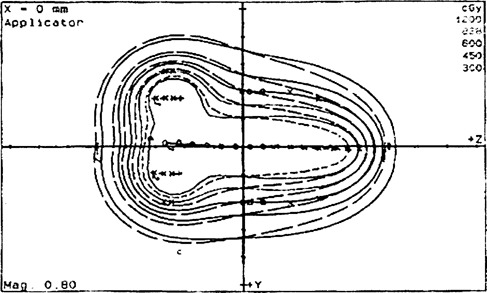
Comparison between PLATO isodose lines optimized (dash lines) and nonoptimized (solid lines), which were 200, 138, 100, 75, and 50% of the prescription point by using 22 dwell positions.

Figure 5 illustrates the comparison between the dose distribution from PLATO optimized and nonoptimized. The distributions are significantly different, specifically near the tip of tandem, where the nonoptimized dose is lower than prescribed, and the ovoid surfaces dose is higher than prescribed. As would be expected from the normalization approach of each program, there is little variation between the two planning systems with respect to the dose to the prescription points.

## CONCLUSION

This study has highlighted the effect of the differences in the calculation algorithm between the older and newer planning systems from Nucletron. While the differences were minimal on the perpendicular to the axis of the source, along the axis they become considerable. In a practical gynecological case, these differences mostly affect the dose to the rectum, since that organ receives the greatest proportion of its dose from rays near the axis. Overall, the PLATO system plan required about 2.5% less integrated reference air kerma than the NPS plan for the same dose to point *A*. In either planning system, optimization is crucial in decreasing dose to bladder and rectal points.
